# Elucidation of Binding Features and Dissociation Pathways of Inhibitors and Modulators in SARS-CoV-2 Main Protease by Multiple Molecular Dynamics Simulations

**DOI:** 10.3390/molecules27206823

**Published:** 2022-10-12

**Authors:** Lei Xu, Liangxu Xie, Dawei Zhang, Xiaojun Xu

**Affiliations:** Institute of Bioinformatics and Medical Engineering, Jiangsu University of Technology, Changzhou 213001, China

**Keywords:** binding features, COVID-19, drug design, M^pro^, MCCS, molecular dynamics simulations

## Abstract

COVID-19 can cause different neurological symptoms in some people, including smell, inability to taste, dizziness, confusion, delirium, seizures, stroke, etc. Owing to the issue of vaccine effectiveness, update and coverage, we still need one or more diversified strategies as the backstop to manage illness. Characterizing the structural basis of ligand recognition in the main protease (M^pro^) of SARS-CoV-2 will facilitate its rational design and development of potential drug candidates with high affinity and selectivity against COVID-19. Up to date, covalent-, non-covalent inhibitors and allosteric modulators have been reported to bind to different active sites of M^pro^. In the present work, we applied the molecular dynamics (MD) simulations to systematically characterize the potential binding features of catalytic active site and allosteric binding sites in M^pro^ using a dataset of 163 3D structures of M^pro^-inhibitor complexes, in which our results are consistent with the current studies. In addition, umbrella sampling (US) simulations were used to explore the dissociation processes of substrate pathway and allosteric pathway. All the information provided new insights into the protein features of M^pro^ and will facilitate its rational drug design for COVID-19.

## 1. Introduction

Coronavirus disease 2019 (COVID-19) caused by severe acute respiratory syndrome coronavirus 2 (SARS-CoV-2), according to the the International Committee on Taxonomy of Viruses (ICTV), is a highly contagious disease [1,2]. As it spreads, the coronavirus can mutate to form the new variants. Recently, the fast-spreading Omicron variant is appeared in different counties with many more mutations [3]. More evidence shows that COVID-19 can affect different neurological symptoms in some people, including smell, inability to taste, dizziness, confusion, delirium, seizures, stroke, etc. Although many countries have issued emergency use authorization for various vaccines for the prevention of COVID-19, vaccines may be less effective against a variant than against the virus that they were initially developed to combat. Fortunately, research can identify which variants of SARS-CoV-2 are more prevalent and prepare vaccines to counter them, which is similar to the process of annual flu vaccines. However, the annual flu vaccines can reduce risk for illness by only 40% to 60% [4], even in years when the vaccine is well matched to the circulating virus strain. To better fight against the COVID-19 infection, we still need one or more diversified strategies that also include new treatments, which may be the safeguard to manage illness attributed to the imperfections in vaccine effectiveness and uptake. The deployment of vaccine is the best measure for controlling the spread of SARS-CoV-2. In addition to vaccine, antiviral drugs are also being used urgently. Remdesivir, a monophosphoramidate prodrug of the nucleoside GS-441524, was the first approved drug with intravenous administration targeting the conserved viral RNA-dependent RNA polymerase (RdRp) [5,6]. Molnupiravir, a small prodrug of the nucleoside derivative N-hydroxycytidine (NHC), is the first approved oral antiviral drug to treat COVID-19. Combination of molnupiravir and nirmatrelvir show synergistic antiviral activity, and Omicron variant remains susceptible for remdesivir, EIDD-1931, molnupiravir and nirmatrelvir [7,8].

For the past one-year, the 3D structures of important enzymes, proteases, and polymerases of SARS-CoV-2 involved in the mechanism of COVID-19 infection have been experimentally resolved, including the spike protein (S-protein) [9], the main protease (M^pro^, or 3C-like cysteine protease) [10], the papain-like protease (PL^pro^) [11], the RdRp [12] and more. Among them, S-protein is a regulatory protein that promotes viral entry of coronavirus into host cells through receptor binding and membrane fusion; M^pro^ and PL^pro^ are indispensable for coronaviral replication; and RdRp is essential for replicating the genome as well as for carrying out transcription. These proteins or enzymes are highly involved in the process of COVID-19 infection, so they are recognized as the key targets for anti-COVID-19 drug discovery. Especially, M^pro^ of SARS-CoV-2, essential for viral replication and transcription, is an attractive antiviral drug target.

An increasing number of three-dimensional (3D) structures of M^pro^ complexed with ligand (s) have been resolved and deposited into the Protein Data Bank (www.rcsb.org) (accessed on 30 July 2020), with over 160 reported structures of M^pro^-inhibitor available (Table S1). These 3D complexes provide an atomic level snapshot of the interactions between M^pro^ and its ligands. In addition, different types of inhibitors [13,14,15,16] of M^pro^ can be observed among these ligands, including the covalent inhibitors, non-covalent inhibitors, and allosteric modulators. For both covalent and non-covalent inhibitors, they bind in the substrate-binding pocket of M^pro^, which is located in the cleft between domain I (residues 8–101) and domain II (residues 102–184). Specially, the covalent inhibitors of M^pro^ bind in the catalytic site cavity (Cys145-His41 catalytic dyad), forming a covalent bond with the catalytic residue-Cys145. On the other hand, the non-covalent inhibitors of M^pro^ are observed to occupy various subsites of the substrate binding site [17]. Last but not least, several allosteric modulators have been reported in recent studies [14,16]. For example, pelitinib (PDB code:7AXM), ifenprodil (PDB code:7AQI), RS-102895 (PDB code:7ABU), PD-168568 (PDB code:7AMJ) and AT7519 (PDB code:7AGA) have been reported to be the allosteric modulators of M^pro^ [16]. Especially, pelitinib, ifenprodil, RS-102895, and PD-168568 bind to the same allosteric binding sites of M^pro^ that formed by Ile213, Leu253, Gln256, Val297, and Cys300 within the C terminal dimerization domain. In addition, AT7519 binds to a second allosteric binding site that formed by Gln107, Gln110, Asn151, Asp153, Thr292, Phe294, and Arg298. Since different compounds of M^pro^ bind to various binding sites in the protein, it is difficult to find a cogent way to characterize the features of M^pro^-ligand binding.

In the present work, we applied molecular dynamics (MD) simulations to systematically characterize the binding features of various binding pockets in M^pro^, utilizing a dataset of 163 representative complexes of M^pro^-ligand. All the results are consistent with the current studies and provide a new insight into the binding features and dissociation pathway of M^pro^, which will facilitate the anti-COVID-19 drug discovery.

## 2. Materials and Methods

### 2.1. M^pro^-Ligand Complexes

The X-ray crystal or cryo-EM structures of the M^pro^ were retrieved from both Uniprot (https://www.uniprot.org/uniprot/P0DTD1#structure, position:3264–3569) (accessed on 30 July 2020) and Protein Data Bank (https://www.rcsb.org) (accessed on 30 July 2020) [18]. Then protein separation and preparation were performed to ensure the accuracy and quality of input data for MCCS [19]. After filtering, we selected 163 complexes of M^pro^-ligand for the further studies, including 44 individual complexes of M^pro^-non-covalent inhibitors, 114 independent structures of M^pro^-covalent inhibitors, and 5 representative structures of M^pro^-allosteric modulators.

### 2.2. Similarity and Clustering

*mccsx* (version 1.1.2, https://github.com/stcmz/mccsx) (accessed on 30 July 2020), a key part of the MCCS implementation, is used to calculate the similarity between every two residue free energy vectors generated by *jdock* using the Pearson correlation coefficient (PCC). The workflow of MCCS is as follow: (1) prepare input PDB file of receptor or ligand; (2) calculate the residue energy contribution by jdock; (3) a full-length protein sequence-based vector is constructed to character the binding feature; (4) a reliable energy contribution vector is employed for protein similarity comparison and clustering [19,20,21].

### 2.3. Biomolecular Electrostatics and Solvation Calculations

In the present work, APBS [22]/PDB2PQR [23] webserver (https://server.poissonboltzmann.org/) (accessed on 30 July 2020) was used for the biomolecular electrostatics and solvation calculations. In brief, PDB2PQR automates many of the common tasks of preparing structures for continuum solvation calculations as well as many other types of biomolecular structure modeling, analysis, and simulation, while APBS (Adaptive Poisson-Boltzmann Solver) solves the equations of continuum electrostatics for large biomolecular assemblages. For the job of PDB2PQR (https://server.poissonboltzmann.org/pdb2pqr) (accessed on 30 July 2020), we adapted the default parameters for the calculations, and the hydrogen bond distance cutoff and hydrogen bond angle cutoff were set as 3.5 Å and 35° to retain more structural data, respectively. Then the output files from PDB2PQR were used for the APBS calculations (https://server.poissonboltzmann.org/apbs) (accessed on 30 July 2020) with the default setting. Finally, the output DX file was downloaded and visualize in PyMol (https://pymol.org/) (accessed on 30 July 2020).

### 2.4. Molecular Dynamics (MD) Simulation and Molecular Mechanics/Generalized Born Surface Area (MM/GBSA) Binding Free Energy Decomposition Analysis

The MD simulations were chosen to explore the binding pattens of two modulators (7AXM and 7AMJ [16]), five inhibitors (5R80, 5RGI, 5RH5, 6W63, and 7JU7) with M^pro^ [14]. The general AMBER force field (gaff) [24] was used for the ligands and the ff99SB force field [25] was used for the proteins. The atomic partial charges of each ligand was derived by fitting the electrostatic potentials using the RESP [26] technique in Amber14. The TIP3P water model [27] was chosen for the explicit water. The complex systems were relaxed by steepest descent methods in 5000 steps. After that, each system was heated and equilibrated in the NVT ensemble from 10 to 300 K over a period of 50 ps. The van der Waals interactions were evaluated within cutoff 12 Å and particle mesh Ewald (PME) [28] method was used for long-range electrostatic interactions. The MM optimization and 100 ns MD simulations for each complex were accomplished in AMBER14.

MM/GBSA free energy decomposition analyses [29,30,31,32,33,34,35,36,37,38,39] were employed to highlight the key residues responsible for the ligand binding applied in the *mm_pbsa* module in AMBER14. The binding interaction of each residue-ligand pair includes four terms (Equation (1): van der Waals interactions (Δ*G*_vdw_), electrostatic interactions (Δ*G*_ele_), the polar part of desolvation (Δ*G*_GB_) and the non-polar desolvation interactions (Δ*G*_SA_).

(1) $$\Delta G_{\mathrm{residue}-\mathrm{ligand}}=\Delta G_{\mathrm{vdw}}+\Delta G_{\mathrm{ele}}+\Delta G_{\mathrm{GB}}+\Delta G_{\mathrm{SA}}$$

The polar part of desolvation (Δ*G*_GB_) was evaluated by Onufriev et al. (*igb* = 2) [40] and the non-polar part of desolvation (Δ*G*_SA_) was determined by SASA with the ICOSA program [41]. Four energy components were evaluated on the basis of 3000 snapshots extracted from the last 30 ns MD trajectory.

### 2.5. Umbrella Sampling (US) Simulations

US, the most classical enhanced sampling method, is used to characterize the dissociation pathways of ligands from its target. Biasing potential were imposed on the reaction coordinate (RC), and the whole RC were divided into a series of continuous window. The RC for 5R80 is the distance between the center of atoms S1, C3, C5, C8 of ligand and the center of main chain carbon atoms of residue His41, Met49, Asn142, Cys145, Glu166, Pro168 and Gln189. The RC for 7AMJ is the center of atoms C3, C11, C14, C21, N1, N2, N3 of ligand and the center of main chain carbon atoms of residue Ile213, Leu253, Gln256, Val7, Cys300 and Ser301. US can derive the system from one thermodynamic state to another.

(2) $$u_{i}=\frac{1}{2}k_{i}{\left(r-r_{i}\right)}^{2}$$

where *u_i_* is the harmonic potential in window *i*. *r_i_* is the reference position in window *i*. *k_i_* represents the elastic constant of restraint potential (5 kcal mol Å^−2^). 5 ns US simulation were carried out for each window to converge the potential of mean force (PMF). The weighted histogram analysis method (WHAM) was used to calculate the PMF curve along RC.

## 3. Results and Discussion

### 3.1. Overview of Binding Sites in M^pro^ Structure

**Figure 1:**
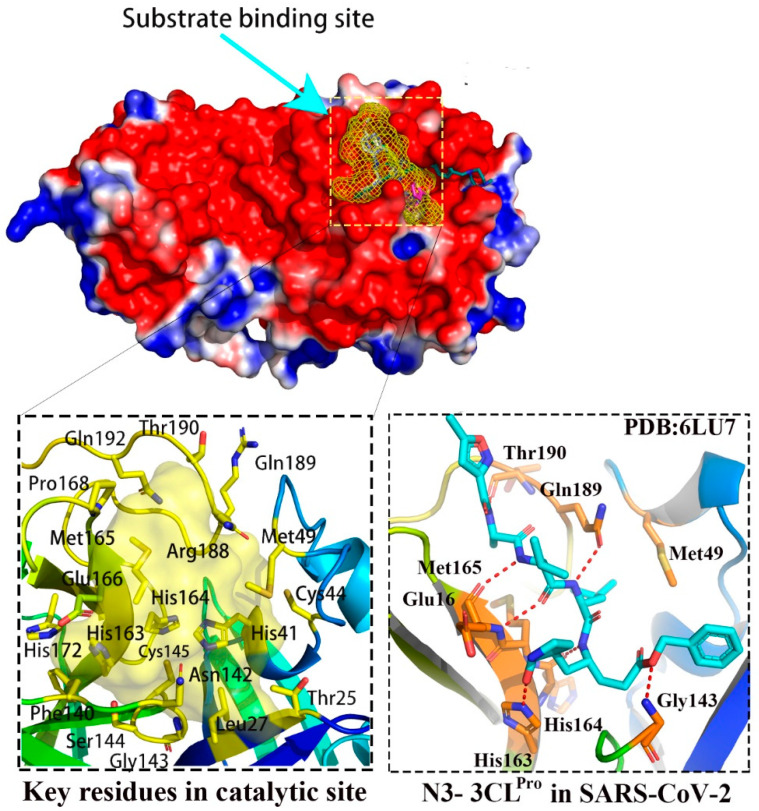
The substrate binding site in M^pro^ structure of SARS-CoV-2.

Up to date, different compounds have been reported to bind to the M^pro^ structure of SARS-CoV-2. Figure 1 shows an overview of binding sites and the related ligands in various binding sites. As shown in Figure 1, the substrate-binding site or the catalytic active site was highlighted in yellow mesh. This binding pocket includes the Cys145-His41 catalytic dyad, and locates in the cleft between domain I (residues 8–101) and domain II (residues 102–184). The key residues involved in this binding pocket include Thr25, Leu27, His41, Cys44, Met49, Phe140, Asn142, Gly143, Ser144, Cys145, His163, His164, Met165, Glu166, Pro168, His172, Arg188, Gln189, Thr190 and Gln192. Different covalent or non-covalent inhibitors have been reported to bind in various subsites of this catalytic active site, including leupeptin (PDB code:6XCH), telaprevir (PDB code:6XQS), narlaprevir (PDB code:6XQT), boceprevir (PDB code:6XQU), adrafinil (PDB code:7ANS), and more. Specially, Cys145 contributes to the binding of covalent inhibitors with the strong covalent bond.

**Figure 2:**
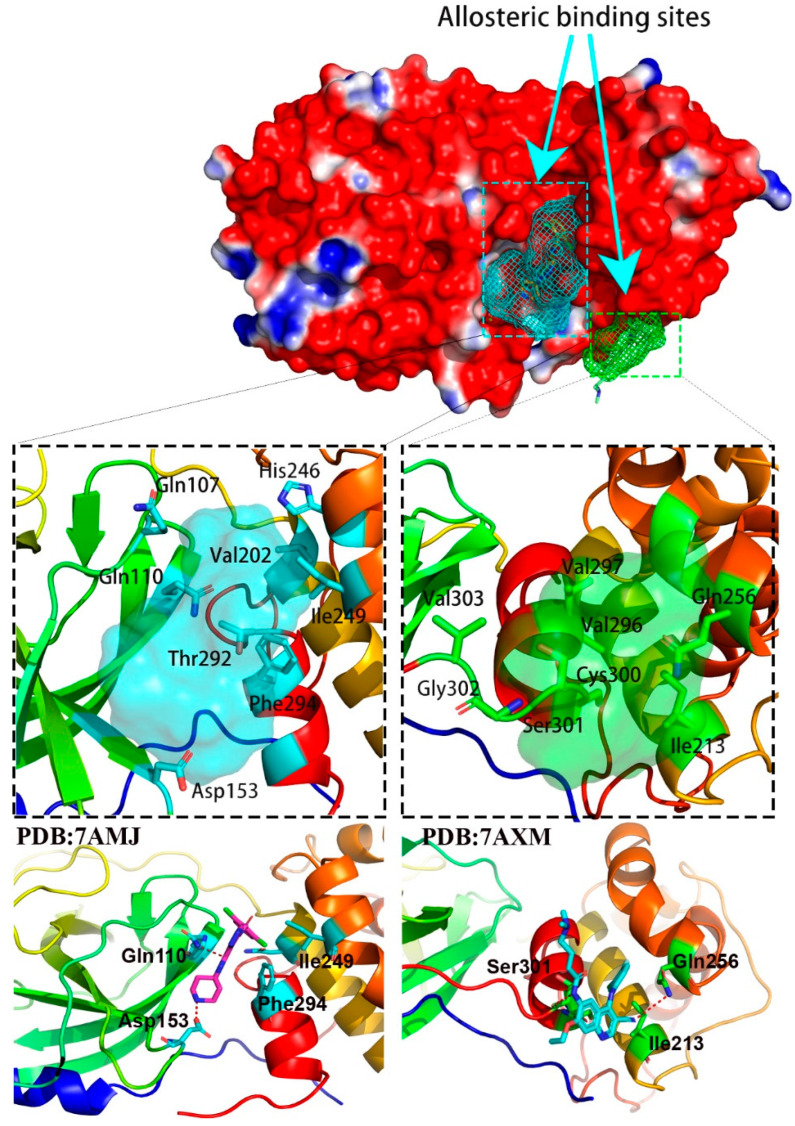
Two allosteric binding pockets in M^pro^ structure of SARS-CoV-2.

Compared to the substrate-binding site, two allosteric binding sites [16] were reported on the opposite surface of M^pro^ structure of SARS-CoV-2, as shown in Figure 2. The first allosteric binding site was highlighted in cyan, which locates between the catalytic domains and the dimerization domain. AT7519, a modulator with moderate activity of M^pro^, is reported to interact with several important residues, including Gln107, Gln110, Asp153, Val202, Ile249, His246, Thr292, Phe294 and more. Specially, Gln110 and Asp153 are observed to form the hydrogen-bond with AT7519, which they may contribute significantly to the recognition of modulators in this pocket. Moreover, a second allosteric binding pocket highlighted in green is close to the first one, as shown in Figure 2. Four modulators that included ifenprodil, pelitinib, RS-102895 and PD-168568 are observed to bind to the same pocket, in which two allosteric modulators including ifenprodil and pelitinib bind to M^pro^ and show antiviral activity against SARS-CoV-2 [16]. As shown in Figure 2, this allosteric binding pocket is hydrophobic and formed by several residues from the C-terminal dimerization domain, including Ile213, Leu253, Gln256, Val296, Val297, Cys300, Ser301, Gly302 and Val303.

### 3.2. Binding Features of Non-Covalent Binders in Substrate-Binding Site of M^pro^

**Figure 3:**
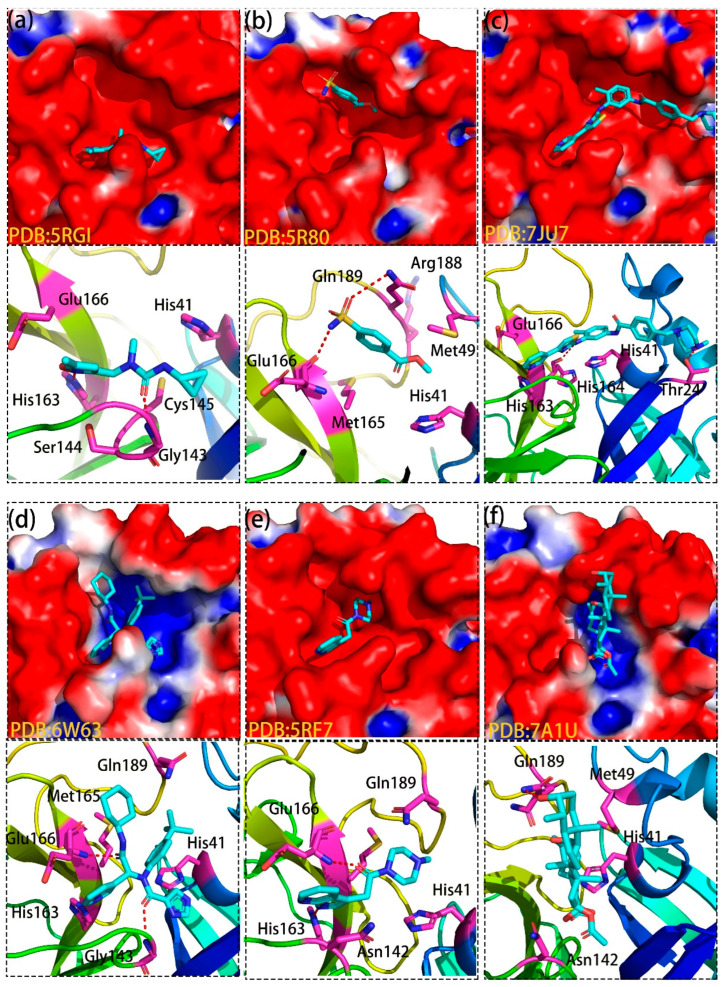
Six non-covalent binders in different substrate-binding subsites of M^pro^. Six binders are randomly selected from 6 groups and observed to bind to different parts of the active site in M^pro^. Bound compounds are depicted as cyan sticks and the surface of M^pro^ is colored by potential on solvent accessible surface. The binding modes of 5RGI (**a**), 5R80 (**b**),7JU7 (**c**), 6W63 (**d**), 5RF7 (**e**), 7A1U (**f**).

First of all, a dataset of 44 complexes of M^pro^ coupled with non-covalent inhibitor were used to characterize the binding features of the catalytic active site. As shown in Figure 3, our results showed that several key residues contributed significantly to the binding of non-covalent inhibitors, including His41, Met49, Phe140, Asn142, His163, Met165, Glu166, Asp187 and Gln189. Specially, His163 and Glu166 were observed to form the strong hydrogen-bonding with ligands, with an average energy contribution of −0.32 and −0.31 kcal/mol.

Six non-covalent binders were selected from different groups using the reported protocol-MCCS [19,20,42], including 5RGI (Group 1), 5R80 (Group 2), 7JU7 (Group 3), 6W63 (Group 4), 5RF7 (Group 5) and 7A1U (Group 6). Figure 3 shows the detailed binding information of six binders in the active site of M^pro^, in which these six compounds showed diverse chemical structures. Interestingly, we observed that these six ligands bind to different parts of the substrate-binding site with distinguished interactions, as shown in Figure 3a–f. For example, inhibitors from Group 1 (Figure 3a) mainly interacted with the Cys145-His41 catalytic dyad, while the ligands from Group 2 (Figure 3b) located in a deep groove formed by Met165, Pro168, Gln189 and Thr190. Moreover, binders from Group 3 (Figure 3c) can extend to domain I (residues 8–101) and form strong interactions with Gly23, Thr24, Thr25 and more. In addition, although the binders from Group 4 (Figure 3d) shared huge similar interactions with that of Group 5 (Figure 3e), and the former ones can form strong interactions with residues from the deep groove where compounds of Group 2 interacted with. Last but not least, the location and interactions of the non-covalent inhibitors from Group 6 (Figure 3f) dramatically differed from that of the other groups, which they mainly interacted with right part of the substrate-binding site that formed by His41, Met49, Gln189, and more.

### 3.3. Binding Features of Covalent Inhibitors in Substrate Binding Site of M^pro^

**Figure 4:**
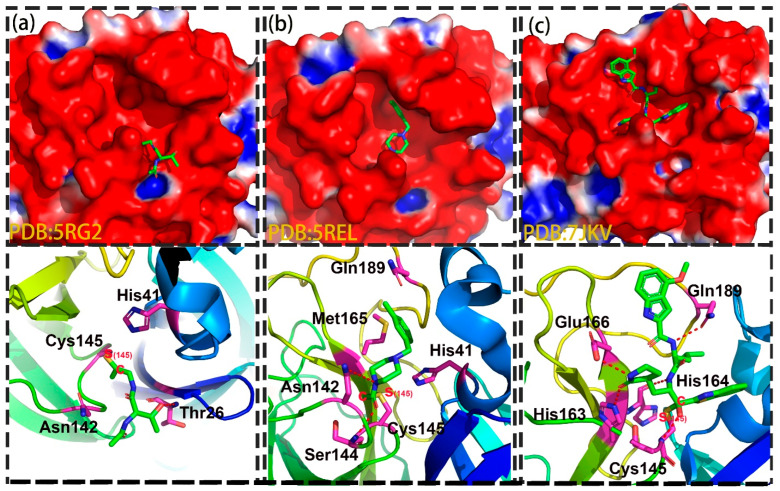
Three representative covalent inhibitors in the substrate-binding site of M^pro^. Three covalent binders are randomly selected from 3 groups. Bound compounds are depicted as cyan sticks and the surface of M^pro^ is colored by potential on solvent accessible surface. The binding modes of 5RG2 (**a**), 5REL (**b**),7JKV (**c**).

Using the same parameters, we then analyzed a dataset of 114 complexes of M^pro^ coupled with covalent binders [43]. We noted that several residues contributed equally to the binding of covalent and non-covalent inhibitors, including His41, Met49, Asn142, His163, Met165, Glu166, Asp187 and Gln189. However, several residues are of importance for the recognition of covalent inhibitors, including Gly143, Ser144 and Cys145, which may attribute to the forming of covalent bond with Cys145. Figure 4 shows the detailed interactions of 3 complexes of M^pro^-covalent inhibitor, including 5RG2 (Group 1), 5REL (Group 2) and 7JKV (Group 3). As shown in Figure 4, all the inhibitors can form strong covalent bond with Cys145. For the binder in Group 1, the key residues included Thr25, Thr26, His41, Asn142 and Cys145, which Thr26 contributed significantly to the ligand binding with a hydrogen-bond. For the inhibitor in Group 2, His41, Asn142, Ser144, Cys145, Met165 and Gln189 were critical for the ligand recognition, in which Asn142 and Ser144 formed strong hydrophilic interactions with the ligand. Last but not least, Ser144, Cys145, His163, His164, Glu166 and Gln189 were the key residues for the ligand binding of inhibitors in Group 3, in which several residues including Ser144, His163, Glu166 and Gln189 were observed to interact with the ligand via hydrogen-bond. More details can be found in Figure 4.

### 3.4. Binding Features of Allosteric Modulators in M^pro^

**Figure 5:**
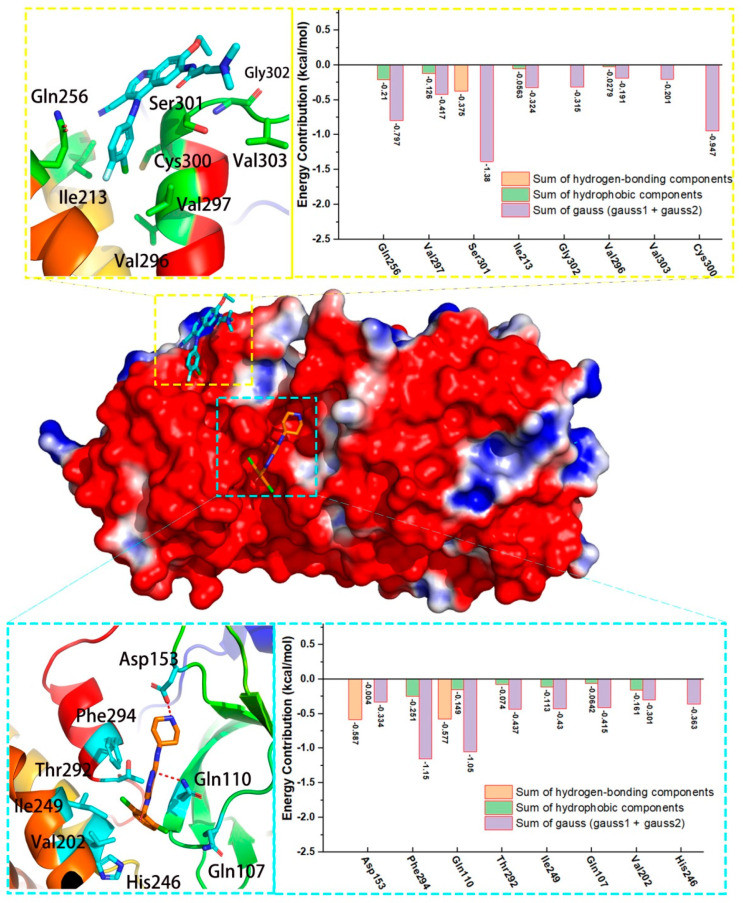
Two representative allosteric modulators in different allosteric sites of M^pro^. The detailed interactions and energy contribution of two allosteric modulators in M^pro^. Bound compounds are depicted sticks and the surface of M^pro^ is colored by potential on solvent accessible surface.

As mentioned in Figure 2, there were two reported allosteric binding sites in the surface of M^pro^, with only 5 available 3D complexes of M^pro^-modulator. As shown in Figure 5, the upper part showed the binding information of modulator in Group 1 (PDB code:7AGA). Our results showed that Ser301 contributed to the binding of modulator with a weak hydrogen-bond (−0.375 kcal/mol), while other four residues contributed to the recognition of the allosteric modulator via hydrophobic interactions, including Ile213, Gln256, Val296 and Val297. We also observed that all the eight residues highlighted in Figure 5 contributed to its binding with gauss energy. In addition, the bottom side of Figure 5 shows the detailed information of allosteric modulator in Group 2 (PDB code:7AXM). We observed that two residues including Asp153 and Gln100 interacted with the modulator with strong hydrogen-bond, with the energy of −0.587 and −0.577 kcal/mol. Five residues that included Phe294, Gln110, Thr292, Ile249 and Val202 formed the hydrophobic interaction with the modulator, and eight residues contributed to the recognition of modulator with gauss energy. More information can be observed in Figure 5.

### 3.5. MD Simulations and Free Energy Decomposition Analyses of M^pro^-Ligands

**Figure 6:**
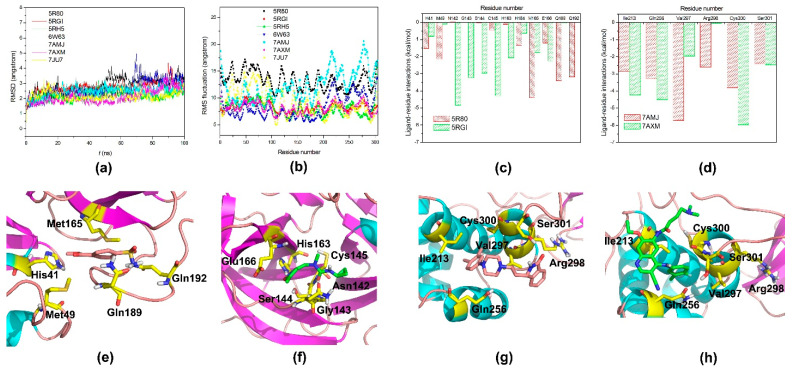
The dynamics, energy and structure comparison of MD simulations. (**a**) The RMSD values of C_α_ atoms with respect to the first snapshots as a function of time; (**b**) The RMSF of backbone atoms versus residue number; (**c**) Binding energy contributions from the key residues of M^pro^ in 5R80 and 5RGI; (**d**) Binding energy contributions from the key residues of M^pro^ in 7AMJ and 7AXM; (**e**–**h**) Comparisons of the averaged structures for 5R80, 5RGI, 7AMJ and 7AXM.

In order to further explore the dynamics interaction patterns and quantitatively evaluate the roles of the key residues, seven complexes of M^pro^-non-covalent inhibitors (PDB code:5R80, 5RGI, 5RH5, 6W63, 7AMJ (modulator), 7AXM (modulator) and 7JU7) were chosen to conduct 100 ns MD simulations and MM/GBSA calculations. The root mean square deviation (RMSD) values for the C_α_ atoms of seven ligands relative to the starting structures during the production phase were shown in Figure 6a, and converged to ~2.84 ± 0.48, 2.74 ± 0.39, 2.18 ± 0.25, 2.62 ± 0.60, 2.48 ± 0.33, 2.12 ± 0.44 and 2.18 ± 0.28 Å. In general, these systems were relative stable during the MD simulations, except for one system (PDB code:6W63) that had a large fluctuation at ~70 ns and then kept stable, in which the other systems had reached equilibrium after ~30 ns. Here, we mainly analyze the MM/GBSA free energy rather than dynamic structure, and according to our previous results [29,30,31,32,33,34,35,36,37,38,39], 100 ns MD simulation for each system is appropriate to calculate binding free energy. The root mean square fluctuations (RMSF) versus the residue number for seven systems were shown in Figure 6b. Since the binding sites of these seven ligands were not exactly the same, the residues showed different fluctuation patterns. The small fluctuations may roughly explain the interactions between some residues and ligands.

MM/GBSA free energy decomposition was employed to obtain ligand-residue interaction spectra to insight into the interaction patterns of M^pro^ with non-covalent binders, Z369936976 from Group 1 (PDB code:5RGI) and Z18197050 from Group 2 (PDB code:5R80), as well as two allosteric modulators, PD-168568 (PDB code:7AMJ) and pelitinib (PDB code:7AXM). The critical residues for ligands recognition were shown in Figure 6c–h, and numerical data were summarized in Table S1. Consistent with the energy contributions calculation of MCCS above, the major favorable contributors of M^pro^ to ligand binding were van der Waals (such as hydrophobic interactions) and electrostatic terms (such as hydrogen-bonding interactions) (Table S2). According to Figure 6c,e,f the key residues of M^pro^ for non-covalent binders from Group 1 and 2 were obviously different, but it was observed that His41, His64, Met165, and Glu166 formed strong interactions with Z369936976 and Z18197050, which may be used for the design of potent binders. Based on the averaged structure from 100 ns MD simulations, Z369936976 can form stable hydrogen-bond interactions with Gly143, Cys145, and His163, and their electrostatic terms for Z369936976 were −3.22 kcal/mol, −1.62 kcal/mol, and −2.08 kcal/mol, respectively. Z18197050 can form stable a strong hydrogen-bond interaction with Gln192, and its electrostatic terms was −2.92 kcal/mol.

As shown in Figure 6d,g,h, the two allosteric modulators, PD-168568 and pelitinib had strong interactions with most of the binding residues in M^pro^, including Ile213, Gln256, Val297, Cys300, and Ser301. The residue Arg298 contributed −2.60 kcal/mol to PD-168568 binding, but only contributed −0.06 kcal/mol to the binding of pelitinib. PD-168568 can form strong hydrophobic interaction with Val297, and its energy contribution was −5.72 kcal/mol, mainly attributing to van der Waals interaction (−5.50 kcal/mol). Pelitinib can also form hydrogen-bond interaction with Arg298, and its electrostatic terms is −3.62 kcal/mol. 93J502 can also form strong hydrophobic interaction with Ile213, and its energy contribution was −4.22 kcal/mol, mainly coming from van der Waals interaction (−4.02 kcal/mol). These structural and energetic analyses may be useful to insight into M^pro^-ligand binding mechanism and design potent and selective M^pro^ binders, which are consistent with the calculations by MCCS.

**Figure 7:**
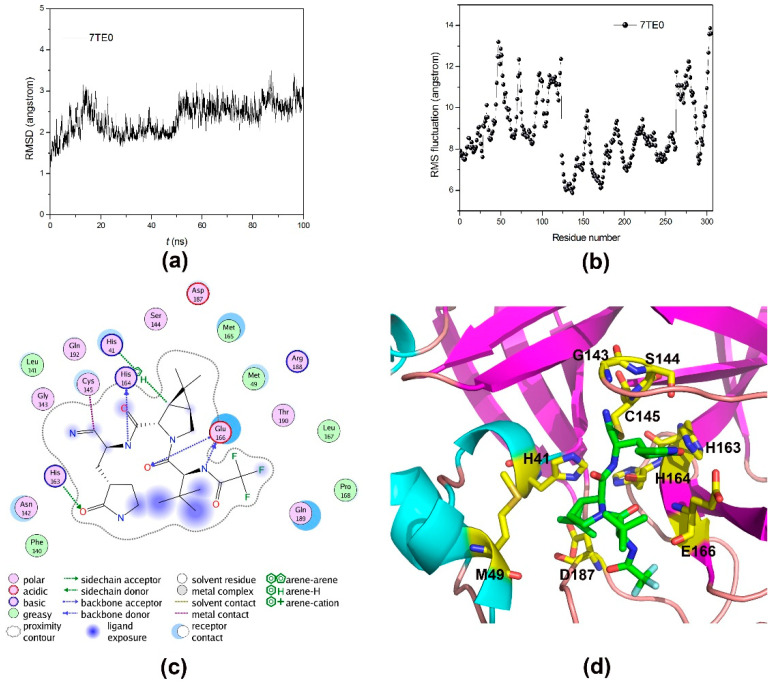
The MD dynamics and structure comparison of M^pro^-nirmatrelvir. (**a**) The RMSD values of C_α_ atoms with respect to the first snapshots as a function of time; (**b**) The RMSF of backbone atoms versus residue number; (**c**,**d**) The 2D and 3D schematic representation of the interactions of averaged structure from MD simulations.

PAXLOVID, the U.S. FDA approved drug, is composed of nirmatrelvir and ritonavir for fighting the COVID-19. Nirmatrelvir is a covalent inhibitor of M^pro^ with high affinity (*K*_i_ = 3.11 nM), and thus we further explore the microscopic binding mode of M^pro^-nirmatrelvir. According to Figure 7a, the M^pro^-nirmatrelvir system reach equilibrium after 50 ns MD simulations and the average C_α_ RMSD is 2.35 ± 0.34 Å. Compared with other systems, the residues showed similar fluctuation patterns. Nirmatrelvir is a tripeptidyl drug, consisting of a C-terminal nitrile warhead and an N-terminal trifluoroacetamide. As shown in Figure 7c,d, the nitrile warhead of nirmatrelvir form a covalent thioimidate adduct with Cys145, and is located in an oxyanion hole including Gly143,Ser144 and Cys145. The trifluoroacetamide group of nirmatrelvir can form van der Waals interaction with Pro168. There are five protein regions that interact directly with nirmatrelvir, including aa40–44, aa45–51, aa140–146, aa163–169 and aa186–192. The carbonyl oxygen atom of β-(S-2-oxopyrrolidin-3-yl)-methyl side chain of nirmatrelvir can form hydrogen-bond interaction with the imidazole of His163. The secondary amine of nirmatrelvir forms hydrogen-bond interaction with carbonyl oxygen atom of His164. The carbonyl oxygen atom of nirmatrelvir can also form hydrogen-bond interaction with secondary amine of Glu166. The tertiary amine of dimethylcyclopropylproline of nirmatrelvir can form cation-π interaction with the imidazole ring of His41 with conserved 40–45 residues. There are other residues forming binding pocket of M^pro^, including Phe140, Leu141, Met165 and His172. Among of them, His172 plays an important role in the binding of β-(S-2-oxopyrrolidin-3-yl)-methyl side chain of nirmatrelvir.

### 3.6. Comparison of the Reaction Coodinate for 5R80 and 7AMJ

**Figure 8:**
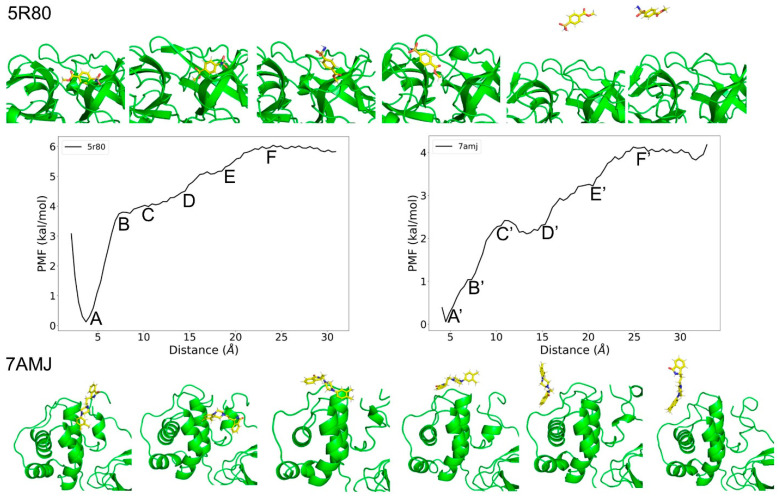
Unbinding processses of ligand RZG (5R80) and ligand RMZ (7AMJ) dissociating from the binding site of M^pro^.

Considering limited sampling capacity of traditional MD simulation, US simulations were chosen to explore the dissociation processes of 5R80 and 7AMJ. As shown in Figure 8, different shape of PMF curve was observed. The higher the curve, the more energy is released during the ligand binding to the M^pro^. It is obvious that the PMF curve for 5R80 is higher than 7AMJ with the increase of the PMF close to 2 kcal/mol. Accordingly, ligand Z18197050 in 5R80, may form stronger affinity with M^pro^ than ligand PD-168568 in 7AMJ. According to Figure 8, the lowest value of PMFs (0 kcal/mol) on the reaction coordinate (point A and A’) denote the bound sates of ligands. Z18197050 in 5R80 and PD-168568 in 7AMJ gradually moves from the substrate pocket and allosteric pocket with the increase of the biasing potential added to ligand, respectively. The reaction coordinate were extended from 0 to 30 Å, representing the ligands 30 Å away from the initial binding site. Compared with the PMF profile of 7AMJ, the PMF curve of 5R80 is much smoother. There is a large barrier (~4 kcal/mol) at ~7.3 Å of reaction coordinate (point B) for 5R80 when the Z18197050 unbinds from the substrate pathway with the conformation of ligand pulled to be nearly flat, and a small barrie (~1 kcal/mol) crosses 5–7 Å for 7AMJ. There is an obvious barrier (~2.4 kcal/mol) at ~11 Å of reaction coordinate (point C’) for 7AMJ when the PD-168568 unbinds from the allosteric pathway with the conformation of ligand pulled to be nearly flat, whereas no rising of the PMFs was observed at ~11 Å of reaction coordinate for 5R80. After ~7 Å of reaction coordinate for 5R80, the rising rate of the PMF is moderated when the Z18197050 gets out of the substrate site, and a large biasing force (~2 kcal/mol at 8–22 Å of the RC) is used to pull the ligand away from the channel to the second maximum. During ~7 Å (point C’) to ~15 Å (point D’) of reaction coordinate for 7AMJ, there is a decrease of the PMF until it reach the local minimum at ~12 Å of the reaction coordinate. After that, similar behavior was found for 7AMJ, and a large biasing force (~2 kcal/mol at 15–25 Å of the RC) is used to pull the ligand away from the channel to the second maximum.

## 4. Conclusions

Although various vaccines have been issued for the emergency use authorization for COVID-19, we still need at least one diversified strategy that also includes new treatment. There is an urgent need to develop new drug(s) against SARS-CoV-2. Among the reported drug targets of SARS-CoV-2, M^pro^ is a key target for the drug discovery of COVID-19. With the advanced experimental technologies, there are over 160 reported complexes of M^pro^-inhibitor available. These inhibitors include the covalent inhibitors, non-covalent binders, and allosteric modulators of M^pro^. Therefore, delineation of protein fingerprint or binding feature of M^pro^ can not only facilitate its structural studies, but also accelerate rational design and development of drugs with high affinities and selectivity.

In the present work, we first systematically characterize the binding features of different binding sites in M^pro^ using the reported MCCS [19,20,42]. For example, the non-covalent binders can bind to different subsites of the substrate-binding pocket, so different residues are of importance for the recognition of the non-covalent binders. Non-covalent binders with potent activity should form strong hydrogen-bonding interaction with His163 and Glu166. Moreover, all the covalent inhibitors can form strong covalent bond with Cys145. Therefore, Gly143, Ser144 and Cys145 may play an important role for the recognition of the covalent inhibitors. In addition, two well-known allosteric binding pockets have been observed in the opposite side of substrate-binding pocket in M^pro^, and these two pockets are close to each other. One allosteric binding site locates between the catalytic domains and the dimerization domain. Gln110 and Asp153 may be the critical residues for the hydrophilic interactions, while Val202, Ile249, His246, Thr292 and Phe294 may contribute to the hydrophobic interactions. Central to the second allosteric binding site is a hydrophobic pocket formed by Ile213, Leu253, Gln256, Val296, Val297, Cys300 and Ser301, Gly302, and Val303. Allosteric modulators with potent activity should form strong hydrogen-bond interaction with Gln100 and Asp153. M^pro^ has been recognized as a significant target for small-molecule and macromolecular/peptidomimetic compounds. Macromolecule and/or covalent inhibitors outperform small-molecule inhibitors in inhibitory potency and selectivity, but suffer from poorer pharmacokinetic properties. Therefore, fragment-based drug design may be a good choice for the design of potential protease inhibitors, incorporating of good fragment form macromolecule compound and small-molecule inhibitor with satisfactory pharmacokinetic properties. A clever combination of inhibitory activity, selectivity and druggability may provide potent drug candidates. The results from MD simulations are consistent with the calculations by MCCS. In addition, US simulations were used to explore the dissociation processes, and we found that the PMF depths of the substrate pathway are much higher than those of the allosteric pathway. With the above computational method, we have new insights into the characterization of M^pro^ coupled with different ligands, which will facilitate the rational drug design and development for COVID-19.

## Data Availability

The data presented in this study are available on request from the corresponding author.

## Supplementary Materials

The following supporting information can be downloaded at: https://www.mdpi.com/article/10.3390/molecules27206823/s1, Tables S1 and S2. The data that support the findings of this study are available within the article and its supplementary material.

## Sample Availability

Sample of the compounds is not available from authors.
