# Supplementary material for: Elucidation of Binding Features and Dissociation Pathways of Inhibitors and Modulators in SARS-CoV-2 Main Protease by Multiple Molecular Dynamics Simulations
Source: Molecules. 2022 Oct 12;27(20):6823. doi: 10.3390/molecules27206823 (PMC9609290; doi:10.3390/molecules27206823)
Supplement: Supplementary file 1 [file molecules-27-06823-s001.zip › molecules-1931827-supplementary.pdf]

Supporting Information

Table S1. The 160 PDB codes used in this manuscript.

PDB ID	Ligand	Idock score (kcal/mol)
5R7Y	A_JFM_1001	-3.63
5R7Z	A_HWH_404	-5.34
5R80	A_RZG_404	-4.83
5R81	A_RZJ_1001	-4.86
5R82	A_RZS_1001	-3.15
5R83	A_K0G_404	-4.77
5R84	A_GWS_1001	-5.75
5RE4	A_SZY_404	-4.19
5RE9	A_LPZ_404	-2.73
5REB	A_T0Y_404	-3.25
5REZ	A_T54_404	-4.02
5RF1	A_T5G_404	-3.71
5RF6	A_NTG_404	-4.57
5RF7	A_T67_404	-6.05
5RFE	A_JGG_404	-3.75
5RG1	A_T9J_404	-3.95
5RGH	A_U0M_404	-3.93
5RGI	A_U0P_404	-5.56
5RGK	A_U0V_404	-2.76
5RGT	A_UHS_405	-2.38
5RGV	A_UGG_1001	-6.24
5RGW	A_UGM_1001	-5.85
5RGX	A_UGP_1001	-6.21
5RGY	A_UGS_1001	-6.28
5RGZ	A_UH1_1001	-6.01
5RH0	A_UH4_1001	-4.1
5RH1	A_UGV_1001	-3.49
5RH2	A_UH7_1001	-6.38
5RH3	A_UHA_1001	-6.95
5RH5	A_UHV_1001	-2.22
5RH6	A_UHY_405	-3.2
5RH7	A_UJ1_405	-3.75
5RH8	A_UHM_405	-4.51
5RHD	A_US7_408	-4.93
6M2N	A_3WL_401	-7.04
6W63	A_X77_401	-8.41
6WNP	A_U5G_401	-1.99
6XFN	ligand	0.56
7A1U	A_FUA_401	-4.98
7AGA	A_LZE_401	-4.05
7ANS	A_RNW_405	-3.86
7AP6	A_RQN_401	-3.89
7AVD	B_S1W_401	-4.83

7AWU	A_S8B_501	-2.45
7JU7	A_G65_401	-0.26
7KX5	A_X7V_401	-8.4
7L5D	A_XNJ_401	2.66
5REJ	A_T1V_404	2.47
5REK	A_T1Y_404	1.93
5REL	A_T2G_404	0.75
5REM	A_T2J_404	2.18
5REN	A_T2V_404	-0.12
5RER	A_T3J_404	0.41
5RES	A_T3V_404	1.77
5RET	A_T47_404	0.21
5REU	A_T4D_404	2.14
5REV	A_T4J_404	3.12
5REW	A_T4M_404	1.14
5REX	A_T4V_404	-1.18
5REY	A_T4Y_404	-0.04
5RFF	A_T6M_404	1.55
5RFG	A_T6V_404	1.3
5RFH	A_T6Y_404	0.54
5RFI	A_T71_404	0.48
5RFJ	A_T7A_404	0.96
5RFK	A_T7D_404	1.84
5RFL	A_T7G_404	1.97
5RFM	A_T7J_404	2.12
5RFN	A_T7P_404	0.86
5RFO	A_T7S_404	1.72
5RFP	A_T7V_404	0.85
5RFQ	A_T7Y_404	1.68
5RFR	A_T81_404	1.26
5RFS	A_T84_405	0.55
5RFT	A_T8A_404	-0.53
5RFU	A_T8D_405	1.87
5RFV	A_T8J_404	1.2
5RFW	A_T8M_405	0.48
5RFX	A_T8P_404	1.84
5RFY	A_T8S_404	1.11
5RFZ	A_T8V_404	2.39
5RG0	A_T8Y_405	1.61
5RG2	A_T9M_404	4.88
5RGL	A_U0Y_404	2.47
5RGM	A_U1D_404	0.34
5RGN	A_U1A_404	1.94
5RGO	A_U1G_404	1.56
5RGP	A_U1M_404	1.18
5RH9	A_UJ4_405	-3.96
5RHA	A_T8M_1001	2.14
5RHB	A_USD_406	4.3
5RHC	A_USA_1001	4.23

5RHE	A_UPD_1001	2.58
5RHF	A_UPJ_405	0.42
5RL0	A_VEG_1001	-1.11
5RL1	A_VEJ_1001	-3.26
5RL2	A_VEM_1001	-2.72
5RL3	A_VEP_405	-4.02
5RL4	A_VEV_405	-3.43
5RL5	A_VEY_405	-3.67
6LZE	A_FHR_405	-1.68
6M0K	A_FJC_405	-1.6
6WTJ	A_K36_401	-1.02
6WTK	A_UED_401	-2.05
6WTT	A_K36_401	-1.51
6XBG	ligand	-2.88
6XBH	ligand	-3.12
6XCH	ligand	-1.21
6XHM	A_V2M_9001	-2.47
6XMK	A_QYS_401	-2.9
6XQS	A_SV6_401	-2.92
6XQT	A_NNA_401	-4.06
6XQU	A_U5G_401	-2.06
6XR3	A_V7G_401	-2.51
6Y2F	A_O6K_502	-2.38
6Y2G	A_O6K_402	-1.21
6YNQ	A_P6N_401	1.08
6YZ6	ligand	0.74
6ZRT	A_SV6_602	-2.53
6ZRU	A_U5G_503	-2.38
7ADW	A_R7Q_504	2.3
7AHA	A_SIN_409	6.1
7AKU	A_RN2_401	-0.79
7AQJ	A_RV8_402	-4.59
7AWS	A_S8E_401	27.6
7AY7	A_S8T_401	5.58
7B3E	A_MYC_407	1
7B83	A_PK8_401	-2.37
7BRP	A_U5G_401	-1.43
7BUY	A_JRY_401	3.11
7C6S	A_U5G_401	-1.6
7C6U	A_K36_401	-0.2
7C7P	A_SV6_401	-2.67
7C8R	A_TG3_401	-3
7C8T	A_NOL_401	-3.44
7C8U	A_K36_401	-0.38
7CBT	A_K36_401	-1.45
7COM	A_U5G_401	-2.2
7CX9	A_GKF_401	4.01
7D1M	B_K36_401	-1.07
7D1O	A_NNA_401	-3.37

7JKV	A_V7G_401	-3.27
7JPZ	A_GHX_401	-2.27
7JQ0	A_VHV_401	-1.59
7JQ1	A_VHJ_401	-2.77
7JQ2	A_VHM_401	-2.57
7JQ3	A_VHP_401	-1.1
7JQ4	A_XM2_401	0.97
7JQ5	A_NOL_401	-2.51
7JYC	A_NNA_401	-2.58
7K40	A_U5G_401	-2.35
7K6D	A_SV6_402	-2.52
7K6E	A_SV6_402	-2.9
7KS5	A_X1Y_503	-3.63
7KYU	A_XC4_501	3.19
7ABU	A_R6Q_401	-3.67
7AGA	ligand	-4.05
7AMJ	A_RMZ_409	-2.9
7AQI	A_QEL_401	-2.84
7AXM	A_93J_502	-2.77

Table S2. Binding free energy contributions of key binding-site residues calculated by the binding energy decomposition for four representative compounds (kcal/mol).

	5R80					5RGI				
	ΔE_{vdw}	ΔE_{ele}	ΔG_{GB}	ΔG_{SA}	ΔG_{bind}	ΔE_{vdw}	ΔE_{ele}	ΔG_{GB}	ΔG_{SA}	ΔG_{bind}
His41	-1.54	-0.34	0.50	-0.12	-1.52	-1.22	0.34	0.38	-0.34	-0.82
Cys44	-0.06	-0.02	0.06	0	-0.04	-0.04	0.04	0	0	-0.02
Thr45	0	-0.04	0.02	0.02	0	-0.02	-0.02	0.02	0	0
Ser46	-0.12	0	0.08	-0.02	-0.04	0.02	-0.04	0.10	0	0
Met49	-1.96	-0.06	0.22	-0.34	-2.14	-0.12	-0.08	0.12	-0.02	-0.10
Asn142	-0.04	-0.02	0.06	0	0.02	-4.80	-0.58	1.26	-0.70	-4.84
Gly143	-0.02	-0.04	0.06	0	0	-1.34	-3.22	1.54	-0.20	-3.22
Ser144	-0.02	-0.02	0.04	0	-0.02	-2.32	-1.58	1.00	-0.06	-2.96
Cys145	-0.40	-0.12	0.16	-0.10	-0.46	-3.24	-1.62	0.84	-0.26	-4.28
His163	-0.18	-0.14	0.18	0	-0.12	-1.62	-2.08	1.72	-0.08	-2.06
His164	-1.80	-0.30	0.92	-0.18	-1.34	-1.26	-0.78	1.48	-0.10	-0.64
Met165	-4.14	-0.32	0.48	-0.21	-4.40	-1.92	-0.22	0.56	-0.16	-1.74
Glu166	-1.22	-1.40	1.66	-0.26	-1.20	-3.20	-0.52	1.96	-0.48	-2.24
Gln189	-4.04	-0.72	1.88	-0.26	-3.40	-0.12	-0.04	0.20	-0.04	-0.04
Gln192	-1.18	-2.92	1.02	-0.06	-3.18	0	-0.02	0.08	-0.06	0
Ile213	0	0	0	0	0	0	0	0	0	0
Gln256	0	0	0	0	0	0	0	0	0	0
Val297	0	0	0	0	0	0	0	0	0	0
Arg298	0	-0.08	0.08	0	0	0	-0.28	0.28	0	0
Cys300	0	0	0	0	0	0	0	0	0	0
Ser301	0	0	0	0	0	0	0	0	0	0

	7AMJ					7AXM				
	ΔE_{vdw}	ΔE_{ele}	ΔG_{GB}	ΔG_{SA}	ΔG_{bind}	ΔE_{vdw}	ΔE_{ele}	ΔG_{GB}	ΔG_{SA}	ΔG_{bind}
His41	0	0	0	0	0	0	0	0	0	0
Cys44	0	0	0	0	0	0	0	0	0	0
Thr45	0	0	0	0	0	0	0	0	0	0
Ser46	0	0	0	0	0	0	0	0	0	0
Met49	0	0	0	0	0	0	0	0	0	0
Asn142	0	0	0	0	0	0	0	0	0	0
Gly143	0	0	0	0	0	0	0	0	0	0
Ser144	0	0	0	0	0	0	0	0	0	0
Cys145	0	0	0	0	0	0	0	0	0	0
His163	0	0	0	0	0	0	0	0	0	0
Met165	0	0	0	0	0	0	0	0	0	0
Glu166	0	-0.08	0.08	0	0	0	0	0	0	0
Gln189	0	0	0	0	0	0	0	0	0	0
Gln192	0	0	0	0	0	0	0	0	0	0
Ile213	-2.60	-0.10	0.32	-0.48	-2.86	-4.02	-0.24	0.68	-0.62	-4.22
Gln256	-3.80	0.02	1.34	-0.84	-3.28	-4.32	-1.86	2.58	-0.92	-4.52
Val297	-5.50	-0.12	0.62	-0.74	-5.72	-1.68	-0.38	0.40	-0.28	-1.96
Arg298	-2.40	-3.62	3.74	-0.30	-2.60	-0.18	-0.10	0.22	0	-0.06
Cys300	-4.04	-0.62	1.46	-0.58	-3.80	-7.16	0.10	2.08	-1.02	-5.98
Ser301	-2.34	-0.72	1.14	-0.48	-2.42	-2.84	-0.20	0.86	-0.32	-2.48
